# The abortion trend after the pronatalist turn of population policies in Iran: a systematic review from 2005 to 2022

**DOI:** 10.1186/s12889-024-19249-4

**Published:** 2024-07-15

**Authors:** Elham Shirdel, Khadijeh Asadisarvestani, Fatemeh Hami Kargar

**Affiliations:** 1 Department of Social Sciences Faculty of Literature and Humanities, University of Sistan and Baluchestan, Zahedan, Iran; 2 Department of Demography and Geodemography, Faculty of Science, Charles University, Prague, Czechia & Department of Social Sciences Faculty of Literature and Humanities, University of Sistan and Baluchestan, Zahedan, Iran; 3 Department of Social Sciences. Faculty of Humanities, Kashan University, Kashan, Iran

**Keywords:** Abortion, Illegal abortion, Legal abortion, Safe abortion, Induced abortion, Population policies, Iran

## Abstract

**Objective:**

Given Iran’s recent shift towards pronatalist population policies, concerns have arisen regarding the potential increase in abortion rates. This review study examines the trends of (medical), intentional (illegal), and spontaneous abortions in Iran over the past two decades, as well as the factors that have contributed to these trends.

**Methods:**

This paper reviewed research articles published between 2005 and 2022 on abortion in Iran. The study employed the PRISMA checklist for systematic reviews. Articles were searched from international (Google Scholar, PubMed, Science Direct, and Web of Science) and national databases (Magiran, Medlib, SID). Once the eligibility criteria were applied, 42 records were included from the initial 349 records.

**Results:**

Abortion is influenced by a variety of socioeconomic and cultural factors and the availability of family planning services. Factors that contribute to unintended pregnancy include attitudes toward abortion, knowledge about reproductive health, access to reproductive health services, and fertility desires, among others. In addition to health and medical factors, consanguineous marriage plays an important role in spontaneous and therapeutic abortion. A higher number of illegal abortions were reported by women from more privileged socioeconomic classes. In comparison, a higher number of medical and spontaneous abortions were reported by women from less privileged socioeconomic classes.

**Conclusion:**

Iranian policymakers are concerned about the declining fertility rate and have turned to pronatalist policies. From a demographic standpoint, this seems to be a reasonable approach. However, the new population policies, particularly, the Family Protection and Young Population Law, along with creating limitations in access to reproductive health services and prenatal screening tests as well as stricter abortion law could potentially lead to an increase in various types of abortions and their associated consequences.

## Introduction

From 2015 to 2019, there was an annual incidence of roughly 121 million unintended pregnancies worldwide, with approximately 61% of them resulting in induced abortion. Although the global rate of unintended pregnancy has declined, the proportion of such pregnancies ending in abortion has increased. Indeed, 45% of these terminations are deemed unsafe, meaning they are carried out in an environment inconsistent with medical standards [1]. Even though preventable, unsafe abortions are among the most important global challenges in terms of public health and human rights. Moreover, they remain a significant cause of mortality and morbidity among women in the developing world [1–4].

Abortions are of different types, including therapeutic (medical), intentional (illegal), and spontaneous abortions. In Iran, unsafe abortion is a highly challenging issue within the context of reproductive health [5]. There are no official statistics on abortions in Iran due to the sensitive nature of this phenomenon and strict abortion laws. It is estimated that 300,000 to 600,000 illegal abortions are performed in Iran every year [6]. Until the enactment of the Family Protection and Young Population Law in 2021, therapeutic abortion was legally authorized with the consent of the mother after a definite diagnosis of fetal anomaly or a life-threatening maternal disease. This could be met if the diagnosis was made by three medical specialists and subsequently confirmed by the legal medicine organization, all before the 19th week of gestation [7–9]. This new law is part of the recent shift toward pronatalist policies in Iran, which started in 2005 [9]. The primary objective of new population policies is to enhance the fertility rate and overall population size. New restrictions have been imposed on access to family planning services, along with stricter rules regarding prenatal screening and abortion [9, 10]. Specifically, this act prohibits the free distribution or financing of contraceptives, the implantation of contraceptive devices, and the promotion of their use. Permanent sterilization of men and women is also prohibited, with female sterilization allowed only in specific cases when the health or life of mothers and pregnant women is in danger. This law also severely restricts access to abortion services [9].

The new population policies, limitations in access to reproductive health and family planning services, and stricter abortion laws have prompted considerable worries. Nonetheless, there is no clear outlook about their impacts on the status of abortion in Iran. Accordingly, the main aim of this study was to examine researches conducted on abortion in Iran from 2005 to 2022. This study illustrates the trend of intentional abortion (illegally performed), spontaneous abortion, and therapeutic abortion, as well as the key factors influencing these types of abortions during this time frame.

## Methods

This systematic review (registration code: CRD42023474372) followed the PRISMA checklist [11].

### Search strategy and selection of articles

Because the primary goal of this study was to clarify the abortion trend in the context of Iran’s new population policies, this review only included studies conducted in Iran between 2005 and 2022. The period chosen for this study was based on the timing of changes in the population policies. While the policies officially shifted towards a pronatalist approach in 2012, discussions about the need for changes in population policies had already begun in 2005. Additional criteria for inclusion were studies written in either English or Persian (Farsi) and published or made available in full text for quality assessment. Studies that were carried out outside of Iran, predated 2005, solely focused on abortions from a medical standpoint, failed to provide the complete text, or were written in languages other than English or Persian (Farsi) were excluded.

The documents were retrieved from national (Magiran, Medlib, SID) and international (Google Scholar, PubMed, Science Direct, Web of Science, CINAHL, and Cochrane Library) databases. The articles were specifically searched using the MeSH vocabulary and the full titles (Prevalence and determinants of abortion in Iran) followed by keywords (therapeutic abortion, spontaneous abortion, illegal abortion, selective abortion, abortion applicants, abortion seekers, induced abortion, legal abortion, aborted embryo, aborted fetus). Initially, 349 records were identified. However, only 42 documents were deemed suitable for the final analysis after they were reviewed per the specified criteria.

### Study selection

**Fig. 1:**
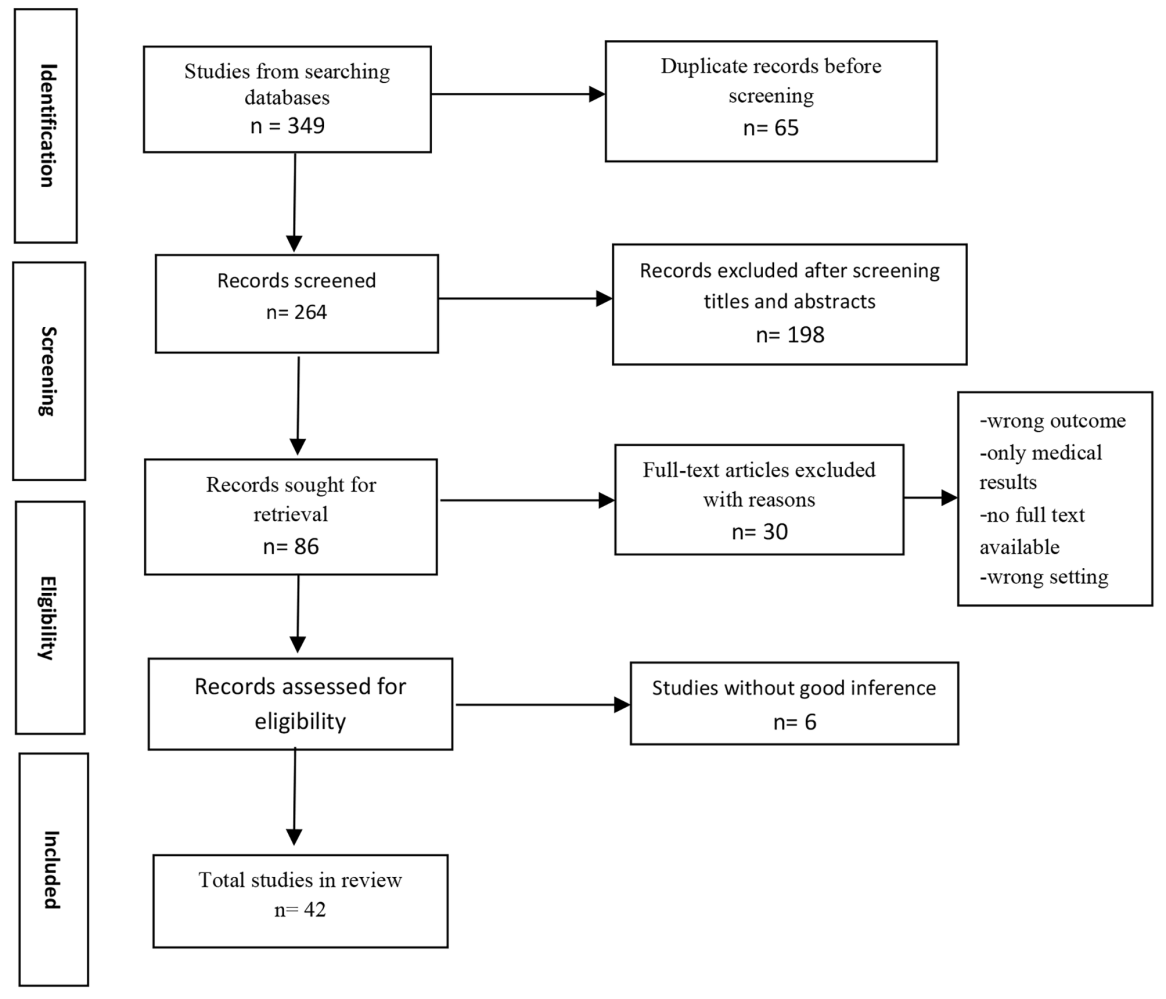
Selection process of the included studies

This systematic review was carried out in accordance with the Preferred Reporting Items for Systematic Review (PRISMA) guidelines (see Fig. 1). The first and second reviewers (KHA and ESH) independently assessed the quality of the included documents, as the quality of a systematic review is based on the quality of the records. Each reviewer screened the titles and abstracts retrieved from the search. If the papers met the inclusion criteria, they were subjected to the reviewing process. If both reviewers disapproved of the title and abstract, the paper was excluded from the study. If there were a discrepancy between the first and second reviewers regarding the selection of a certain document, the third reviewer (FHK) made instead of would be brought in to make the final decision on whether to include the paper in the review.

### Data extraction

A third reviewer (FHK) extracted data from submitted articles, following PRISMA guidelines. In addition, the first reviewer (KHA) and the second reviewer (ESH) checked the extracted data for potential errors to ensure the information’s quality.

The data extracted from each article included the following: author/authors, year of publication, study population/participants, study context (which province, city, or rural area in Iran), sample size, study design, and research outcomes. Finally, the data were presented in a summary table.

### Risk of bias

In order to reduce bias, the quality of the articles was evaluated by two independent reviewers using a checklist of inclusion and exclusion criteria and also based on the Newcastle-Ottawa scale. The Newcastle-Ottawa scale is a tool used to evaluate the quality of non-randomized studies in a systematic review. Using this tool, each study was examined in terms of the selection process of the study groups and the research method, and stars were given for scoring. Studies with less than four stars were excluded from the study. The process of giving stars to the studies was done by two reviewers (ESH and KHA). The studies were examined by the third reviewer (FHK), where there was a disagreement between the two reviewers, and finally the final summary was made.

### Strategy for data synthesis

A PRISMA flowchart was developed to aid in process transparency based on how the search results were presented and how many studies were included in the review. In order to summarize the findings of the included articles, a narrative synthesis of the key findings of the studies was presented in answer to the review questions. The factors related to non-medical abortions were collected and reported from the relevant studies. Furthermore, a summary table summarizing the key elements of the research, such as study settings, participants, and abortion-related variables, is supplied.

## Results

Nine of the 42 studies included in this study [12–20] examined people’s attitudes toward abortion. Fifteen studies [7, 21–34] investigated the factors associated with spontaneous abortion and therapeutic abortion, while eighteen studies [35–55] focused on illegal abortion (see Table 1). In the conducted studies, women aged 25 to 35 exhibited the highest rate of intentional abortions [37, 43, 46–49]. Moreover, the mean age of mothers undergoing therapeutic abortions fell within the range of 28 to 32 years [21, 22, 28].

### The role of socio-cultural factors

Iranian society holds differing views on abortion, ranging from complete rejection to acceptance [12]. However, most Iranians hold a neutral or negative stance, believing that the appropriateness of a decision should be contingent on the specific circumstances [15, 16, 20]. Moreover, studies have shown that women tend to hold more positive attitudes toward abortion compared to men and younger individuals [14, 15, 17]. Compared to married people, unmarried and divorced people hold a more favorable view of abortion [14, 15]. A study found that Fars people have a more favorable attitude toward abortion compared to other ethnic groups [14]. The partner’s attitude is a contributing factor in the occurrence of illegal abortions among women. The findings indicate that some women underwent abortion due to pressure from their partners [38, 47, 49].

The findings also showed that religious affiliation has correlation with attitudes toward abortion and guilt following an abortion [35, 38]. Religiosity and religious adherence decline the positive attitude toward abortion [13–15, 17, 18] and rate of intentional abortion [35–37, 39, 40, 42, 44, 45, 47, 48, 52, 53]. Some women conceal abortion from people around them due to religious and cultural reasons, as well as the social stigma associated with engaging in an unlawful activity [53, 54]. 

The shift in social norms regarding childbearing and the ideal number of children fosters a favorable attitude toward abortion [14, 15]. It increases motivations for intentional abortion, particularly in the event of unintended pregnancy [38, 51]. The incidence of illegal abortion has a significant relationship with pregnancy after achieving the desired number of children [37, 40–42, 46, 50, 51, 53]. There is a higher incidence of spontaneous and therapeutic abortions among women from less privileged socioeconomic classes. However, illegal abortions are more prevalent among women belonging to higher socioeconomic classes [13, 15, 16, 24, 27, 28, 33, 35, 37, 40–42, 46, 48, 53]. Economic and livelihood problems are among the factors that diminish women’s desire to keep the baby and, consequently, increase the abortion rate [37, 38, 44, 49, 51]. Family conflicts, particularly those involving couples, have been identified as factors that contribute to women’s inclination to retain their baby in the event of an unintended pregnancy [41, 49, 51]. Another factor to consider is nontraditional pregnancy, which refers to an unintended pregnancy during dating or engagement, leading to illegal abortion [41].

### The role of health-related factors and unintended pregnancies

Pregnancy health encompasses the well-being of both the mother and baby throughout pregnancy. Factors such as uterine defects, maternal diseases, and the fetal’s genetic abnormalities increase the risk of spontaneous abortion [33, 55]. When it comes to therapeutic abortion, the primary reasons are disorders such as thalassemia and nervous system defects in the baby and cardiovascular problems in the mother (55, 24, 27, 23, 22, 29, 105, 111, 7, 30, 25). Some studies have noted that the type of marriage influences the rate of spontaneous and therapeutic abortions. The significance of consanguineous marriage in the incidence of spontaneous abortion cannot be understated, as it leads to a higher likelihood of chromosomal mutations and genetic defects [27, 32, 34]. The findings of a study by Aghakhani et al. (2017) in Urmia revealed that over 20% of legal abortions performed due to baby disorders were associated with consanguineous marriages [27].

Unintended pregnancy is the main reason for intentional (illegal) abortion [36, 39, 44]. Women who experience an unintended pregnancy within two years of their previous childbirth are more likely to seek abortion compared to women who have pregnancies at appropriate time intervals [50, 51]. Inadequate reproductive health knowledge and insufficient access to reproductive health services are among the major determinants of unintended pregnancies [35, 40, 42, 46, 48, 50, 53].

**Table 1:** Characteristics of the included studies and summary of findings

Attitudes toward abortion				
Authors	Year of research/Year of publication	Study design	Statistical population/**City**	Results
Chinichian & Poorreza	2002/2004	qualitative	50 women of reproductive age in one of Tehran’s central neighborhoods**/ Tehran**	Women’s abortion beliefs range from absolute prohibition to inevitable and accepted, with women being more interested in modern methods of abortion than traditional methods.
Sarayi & Roshanshomal	2006/2012	quantitative	300 women of reproductive age in Tehran**/ Tehran**	The level of religious adherence, the attitude of people around the mother toward abortion, unintended pregnancy, and social class are factors influencing women’s attitudes toward abortion.
Jarahi et al.	2009/2013	quantitative	**480 newly married women/ Mashhad**	The majority of women were opposed to abortion. One of the most important factors influencing positive attitudes toward abortion was a lack of knowledge and awareness of abortion complications. Factors influencing positive attitudes toward abortion included high education, divorce, young marriage age, economic problems, and the use of contraceptives.
Ranjbar et al.	2010/2014	quantitative	400 women referring to health centers**/ Kermanshah**	Most women had neutral attitudes toward abortion. Having a positive attitude toward abortion was linked with higher education, employment, and a history of abortion.
Movahed et al.	2013/2014	quantitative	600 people aged 18 to 29**/ Shiraz**	One of the most important factors influencing positive attitudes toward abortion is adherence to religious values and beliefs. Women were more positive about abortion than men.
Forootan & Sadeghi	2014/2017	quantitative	4267 men and women aged 15 and above**/ Esfrain, Ahvaz, Babolsar, Bojnord, Khoramabad, Saqez, Kamiyaran, Gonbadkavos, Mahmoudabad and Hamadan**	Nearly two-fifths of those surveyed had a favorable attitude toward abortion. Younger, more educated, unmarried women were more supportive of intentional abortion than men were. In addition to the ideal number of children, gender attitudes toward labor division and religious beliefs are important factors in attitudes toward intentional abortion.
Mousavi et al.	2014/2018	quantitative	450 women of reproductive age**/ Hamedan**	Most of the participants had a negative attitude towards abortion. Women with higher education and employed women had a more positive attitude toward abortion.
Rahimi et al.	2015/2016	quantitative	146 medical students of Babol**/ Babol**	Religious beliefs influence students’ attitudes toward abortion, and higher education and employment both play a role in positive attitudes toward intentional abortion.
Kheialo et al.	2020/2021	quantitative	384 students of Tehran University**/ Tehran**	Younger women, singles, and divorcees have a more positive attitude toward abortion. Religion and child value both have a significant negative relationship with abortion. Participation in leisure activities, a positive attitude toward extramarital relations, and gender equality all have a significant positive relationship with abortion attitudes. Religion is the most important factor in explaining the tendency to intentional abortion.
**Illegal abortion**				
Authors	Year of research/ Year of publication	Study design	Statistical population/ **City**	Results
Shahbazi et al.	2006/2008	qualitative	27 women with a history of intentional abortion/ **Tehran and Karaj**	The significance of friends, family, and the treatment team’s views on abortion was highlighted. Women who had abortions did not hold strong views on the Shari’a prohibition on abortion.
Erfani	2007/2008	quantitative	73,200 married women aged 15 to 49 /**28 provinces**	Cities with higher levels of religious belief had fewer intentional abortions, and access to modern contraceptives and family planning education were effective in reducing intentional abortions, particularly in cities.
Chinichian et al.	2007/2007	qualitative	36 group interviews with women from different ethnicities in different regions and 53 interviews with experts involved in intentional abortions/ **Iran**	Unintended pregnancy in unfavorable economic circumstances is the most common reason for intentional abortion, and religion is the most common reason for opposing an abortion.
Hosseini-Chavoshi et al.	2005/2012	quantitative/qualitative	5526 married women between 15 and 54 years old; 40 in-depth interviews/ **Tehran, Gilan, Isfahan, Yazd**	Women with higher education, older age, and more than two children have more abortions. Abortion decreases as religiosity increases. Women hide it from others because of the Shariah and legal prohibitions on abortion. They feel guilty and depressed after having an abortion.
Erfani	2009/2011	quantitative	2934 married women aged 15 to 49/ **Tehran**	Intentional abortion is associated with high education levels, having two children, working women, low religiosity, and low income. The majority of abortions are performed on women who use pills and condoms to prevent pregnancy. Abortion rates were highest among women aged 30 to 34.
Nikpour et al.	2011/2013	quantitative	513 women with symptoms of miscarriage were referred to hospitals in the south of Tehran/ **Tehran**	Women who had abortions were between the ages of 25 and 31. Assuming to have “enough” children, using inappropriate methods of preventing pregnancy, and increasing education were factors influencing intentional abortion.
Veisi & Zanganeh	2011/2013	quantitative	91 women who had an intentional abortion during the past 5 years/ **Kermanshah**	Unintended pregnancy is caused by traditional methods such as withdrawal, and women lack standard prevention methods. Assuming the number of children was enough and consecutive pregnancies were the primary reasons for abortion.
Ranji	2012/2012	quantitative	2705 ​​married women between 15 and 45 years old who had files in health centers/ **Urmia**	Induced abortion is associated with education level, family income, religion, ethnicity, number of children, and age at marriage. Non-medical providers performed one-third of all abortions. The most common reasons for abortion were a desire to stop or postpone childbearing and family economic problems.
Ebtekar et al.	2011/2013	qualitative	5 Kurdish women who had an intentional abortion a year ago/ **Sanandaj**	Economic issues, pressure from the spouse, and concerns about society’s opinion were among the reasons given by these women. The spouse played a significant role in the decision of the wife for an abortion. Women who had abortions experienced guilt and regret in addition to pain and bleeding.
Motaghi et al.	2012/2013	qualitative	72 interviews with women of different ethnicities (including Fars, Gilak, Mazandarani, Arab, Azerbaijani, and Lor) who had unsafe abortions, women who did not abort despite unintended pregnancy, gynecologists, and midwives/ **Tehran and Shahrood**	Religious beliefs have been the primary factor in refusing abortion, even in the case of an unintended pregnancy.
Nejati Hatamian	2012/2014	qualitative	28 women with a history of intentional abortion/ **Tehran**	The significance of the spouse’s role and the attitude of those around her in the decision to have an abortion, the age of women between 26 and 30 years at the time of abortion, and religious attitudes were mentioned as impediments to abortion
Abdoljabbari et al.	2013/2016	quantitative	369 women who had an intentional abortion in the past 10 years/ **Tehran**	The mean age of women who underwent abortions was 26 years. Family disputes and financial difficulties were two of the leading causes of abortion. One of the most influential inducers of abortion was the spouse.
Mohammadi et al.	2013/2014	qualitative	23 women applying for intentional abortion/ **Tabriz**	The trend toward having few children, economic challenges, familial conflicts, the disruption caused by childbearing on employment, and the short interval between children were discussed.
Nasrabadi	2014/2017	qualitative	50 women with intentional abortion/ **Tehran**	Economic difficulties, the belief that two children are sufficient, having children outside the norm, family conflicts, and the employment status of women contributed to female abortion.
Rezaei & Partovi	2016/2018	quantitative	360 married women aged 15 to 49/ **Mahabad**	Educated, employed women of high socioeconomic status are at a greater propensity to perform intentional abortions.
Erfani	2014/2016	quantitative	**In the first and second stages, there were 2,934 and 3,012 married women aged 15–49, respectively/ Tehran**	Women who were employed possessed a higher level of education, were non-religious, and had two children or fewer had a higher incidence of abortions. The majority of women had employed non-modern techniques to avert pregnancy.
Erfani	2014/2021	quantitative	3012 married women aged 15 to 49/ **Tehran**	Intentional abortions were more prevalent among the following demographic groups: women in their final years of childbearing, employed women, women who intended to pursue further education, women who identified as having low levels of religiosity, women who had two children, and women who expressed no desire for additional children. Women used non-modern contraceptive methods.
Gholami	2020/2021	quantitative	214 women with unintended pregnancy/ **Kazerun**	The relationship between religious beliefs and intentional abortion is significant in cases of unintended pregnancy.
**Spontaneous and therapeutic abortion**				
Authors	Year of research/ Year of publication	Study design	Statistical population/ **City**	Results
Hassanzadeh Nazarabadi et al.	2004/2006	quantitative	354 couples with a history of spontaneous abortion/ **Mashhad**	Chromosomal abnormalities are more common in consanguineous marriages.
Sadr et al.	2004/2006	quantitative	1101 abortion licenses issued in forensic medical centers of Iran/ **whole country**	63% were attributed to fetal problems. Nearly half of the mothers were aged 20 to 30.
Sayedoshohadaie et al.	2007/2011	quantitative	Eighty-five women who sought abortions at forensic medicine/ **Sanandaj**	Fetal encephalitis was the prevailing fetal-related cause, while maternal cardiovascular diseases were the most prevalent maternal causes, accounting for 58% of abortions. The 30–35 age group had the most applicants.
Rostam Nejad et al.	2007/2010	quantitative	80 women applying for abortion/ **Ardabil**	Permits were issued for 70%. The causes of 87% were fetal brain disorders. The mean age was 28 years.
Naeeji et al.	2008/2012	quantitative	774 women applying for an abortion/ **Tehran**	71% of requests were approved. The primary cause of abortion was fetal encephalopathy.
Soleymanpour et al.	2014/2017	quantitative	629 women applying for abortion with fetal causes/**Esfahan**	The biggest problem of the fetus was brain disorders. During these four years, 830 licenses were issued.
Astaraki et al.	2013/2015	quantitative	205 women applying for abortion/ **Khorramabad**	The permit was issued for 70% of applicants. Those aged 24–34 years comprised the majority. The most common reason for abortion was fetal brain disorders, and among maternal problems, cardiovascular diseases were the primary reason for obtaining an abortion permit.
Aghakhani et al.	2015/2018	quantitative	80 women applying for abortion/ Urmia	The mean age was 30 years. 90% of abortions are due to fetal problems. 22% had a consanguineous marriage.
Shams Ghahfarokhi	2014/2018	quantitative	484 women with consanguineous marriages and 124 women with consanguineous marriages/ **Isfahan**	Consanguineous marriage increases the risk of spontaneous abortion.
Mousavi et al.	2015/2018	quantitative	550 women applying for abortion with fetal causes/ **Khorasan Razavi**	The most common cause was related to fetal brain disorders. The mean age of mothers was 28 years.
Amini & Yari Nasab	2017/2021	quantitative	517 women applying for abortion/ **Boyer Ahmed**	85% of applicants were granted permission. 92% of abortions were due to fetal defects and thalassemia major.
Sarani et al.	2017/2019	quantitative	200 women with a history of spontaneous abortion/ **Zahedan**	Lack of health literacy causes pregnancies that lead to spontaneous abortions. 55% of women with spontaneous abortion were illiterate.
Alizadeh Mohajer et al.	2017/2020	quantitative	203 women applying for abortion/ **Ilam**	Permits were issued for 52% of applicants. 80% of applications were due to fetal problems.
Fatemi & Akbari	2019/2020	quantitative	305 women with abortion permits/ **Lorestan**	The most common cause of abortion was fetal problems. The mean age of mothers was 31 years.
Sharifi et al.	2010/2019	quantitative	428 women applying for abortion/ **Kermanshah**	The most common reasons for abortion are fetal problems, brain abnormalities, and cardiovascular problems of the mother.

## Discussion

The main goal of this study was to investigate the trend of abortion and the primary factors contributing to it in Iran after the turn in population policies from antinatalist to pronatalist. Accordingly, this systematic review analyzed 42 studies published on therapeutic (medical), intentional (illegal), and spontaneous abortions between 2005 and 2022.

The findings indicate that abortion is affected by a complex set of interrelated factors. The most important factor in illegal abortion is unintended pregnancy [1, 56], which is one of the major health concerns in Iran [1, 57–59]. Critics of Iran’s new population policies believe that the limited access to contraceptives and reproductive health knowledge will increase the number of unintended pregnancies and illegal abortions. Put simply, when most couples prefer to have one or two children, imposing restrictions on family planning services and prenatal screenings not only fails to boost the fertility rate but also leads to a rise in abortion rates and its associated consequences [9, 60]. Despite the strict abortion rules that have been in place since 1979, unofficial estimates show that many women undergo illegal abortions, often without any attention to legal consequences [9, 39, 61]. A study reported that from 2015 to 2017, 21,477 cases in Iran were referred to the legal medicine centers to obtain abortion permission, but 27.29% of these cases were rejected. Most of these rejections (25.8%) were cases with major anomalies but their gestational age had exceeded 19 weeks [62]. This study shows that even before the enactment of the new population law in 2021, there were shortcomings in health reproductive and family planning programs.

Indeed, limiting access to prenatal screening and implementing strict abortion laws may seem to decrease the number of medical abortions. However, in reality, it can lead to an increase in illegal and spontaneous abortions [9, 57, 63]. An overview of the abortion situation around the world reveals that the rate of unsafe abortions is higher in countries with stricter abortion laws [64]. As an example, Brazil has the highest estimated frequency of abortions in the world, while it has the most punitive laws for illegal abortions [65]. There are concerns that Iran may be repeating mistakes similar to the strict pronatalist policy implemented in Romania in 1966. This policy banned abortion (with some exceptions) and greatly limited access to contraception in order to increase the fertility rate. The outcome was a significant increase in unintended pregnancies, unsafe abortions, and maternal morbidity [9, 57, 63].

The findings of this study showed that illegal abortions were reported to a greater extent by women from more privileged socioeconomic classes and less religious groups. In contrast, spontaneous abortions and therapeutic abortions were reported to a greater extent among women from less privileged socioeconomic classes [9, 62]. It is believed that the new limitation in access to family planning services can negatively affect couples who are living in rural areas rather than urban areas because before the new law, many of them relied on the free provision of contraception by health centers [9].

While further investigation is needed to fully understand the disparities between socioeconomic classes, the impact of these disparities on abortion rates underscores the significance of considering socioeconomic and cultural factors when developing population policies. So, it is important for policymakers to consider that Iran is a vast country with different economic, social, and cultural groups. While there are certain similarities among these groups [9, 66], they have differences in their fertility desires and attitudes towards population policies and abortion [67]. As a result, implementing a uniform population policy that ignores these differences may lead the country down the wrong path [9]. Policymakers should consider that fertility is a multidimensional issue and Iran’s dramatic fertility decline over the recent decades can be seen as a response to the wider global transition towards smaller family size [68]. So, it is impossible to draw a simple cause-effect link between policies and fertility change [9, 69]. The findings of this study showed that economic difficulties and family conflict are among the contributing factors to illegal abortions, thus, the government can support such families instead of the stricter abortion law.

Last but not least, when it comes to abortion, the main focus is on women. Men play a significant role in women’s decision-making process regarding abortion, and their involvement can impact the way women seek care [70]. Based on the findings, some women have had abortions under the pressure of their partners. Thus, designing effective reproductive health policies requires considering the role of men and improving their participation in this regard.

In sum, focusing on social and economic factors affecting fertility intentions would be better than violating the reproductive rights and freedoms defined in the 1994 International Conference on Population and Development (ICPD) Programme of Action [9, 71] public health planners can improve their strategies to reduce the number of therapeutic (medical) and spontaneous abortions and support families to have healthy children and obtain their desired number of children. Increasing access to family planning and abortion services as well as improving reproductive health knowledge can decline unintended pregnancies, abortions, and their costs both for families and society.

### Strengths and limitations

This study reviewed all studies conducted on different types of abortion during the past two decades in Iran. It provided a clearer picture of abortion and its major contributors following Iran’s shift toward pronatalist population policies. One of the primary limitations of this study is the absence of precise data on the rate of abortion and its fluctuations during the period under investigation. Furthermore, because causality cannot be established using the systematic review method and observational studies, we only discussed the factors that contribute to abortion.

### Further research

Most studies on abortion in Iran have been conducted in larger, more developed cities. In addition, no studies were found regarding abortion among sexually active single women, immigrants, and women with disabilities. It is crucial to conduct thorough studies to assess and monitor the effects of recent population policies on all types of abortions across different groups of society.

## Conclusion

This study tried to illustrate the trends of therapeutic (medical), intentional (illegal), and spontaneous abortions in Iran after turns to pronatalist population policies as well as clarifying their main contributing. The findings of this study showed that abortion is a complicated issue which is affected by a complex set of socio-economic factors, fertility desires, accessibility of family planning services and population policies. In spite of some similarities, each type of abortion has its special contributing factors. Accordingly, Iranian policymakers should be aware of the possible impacts of new population policies on different types of abortions. Tightening abortion laws, restricted access to family planning services and prenatal screening tests may inadvertently lead to an increase in all types of abortions.

Population policies encompass more than just engineering the population size. They also involve prioritizing and enhancing population health, as well as upholding reproductive health rights. These aspects are crucial and interconnected components of population policies.

## Data Availability

All data generated or analysed during this study are included in this published article.
